# Highly Toxic Ribbon Worm *Cephalothrix simula* Containing Tetrodotoxin in Hiroshima Bay, Hiroshima Prefecture, Japan

**DOI:** 10.3390/toxins5020376

**Published:** 2013-02-20

**Authors:** Manabu Asakawa, Katsutoshi Ito, Hiroshi Kajihara

**Affiliations:** 1 Graduate School of Biosphere Science, Hiroshima University, Higashi-Hiroshima, Hiroshima 739-8528, Japan; 2 National Research Institute of Fisheries and Environment of Inland Sea, Fisheries Research Agency, Maruishi 2-17-5, Hatsukaichi, Hiroshima 739-0452, Japan; E-Mail: katsuit@affrc.go.jp; 3 Faculty of Science, Hokkaido University, Sapporo, Hokkaido 060-0810, Japan; E-Mail: kazi@mail.sci.hokudai.ac.jp

**Keywords:** ribbon worm, *Cephalothrix simula*, Hiroshima Bay, tetrodotoxin, HPLC-FLD, ESI-MS, IR, NMR, GC-MS

## Abstract

In 1998, during a toxicological surveillance of various marine fouling organisms in Hiroshima Bay, Japan, specimens of the ribbon worm, *Cephalothrix simula* (Nemertea: Palaeonemertea) were found. These ribbon worms contained toxins with extremely strong paralytic activity. The maximum toxicity in terms of tetrodotoxin (TTX) was 25,590 mouse units (MU) per gram for the whole worm throughout the monitoring period. The main toxic component was isolated and recrystallized from an acidified methanolic solution. The crystalline with a specific toxicity of 3520 MU/mg was obtained and identified as TTX by high performance liquid chromatography (HPLC)-fluorescent detection (FLD) (HPLC-FLD), electrospray ionization-mass spectrometry (ESI-MS), infrared (IR), nuclear magnetic resonance (NMR) and gas chromatography–mass spectrometry (GC-MS). The highest toxicity of *C. simula* exceeded the human lethal dose per a single worm. A toxicological surveillance of *C. simula* from 1998 to 2005 indicated approximately 80% of the individuals were ranked as “strongly toxic” (≥1000 MU/g). Forty-eight percent of the specimens possessed toxicity scores of more than 2000 MU/g. Seasonal variations were observed in the lethal potency of *C. simula*. Specimens collected on January 13, 2000 to December 26, 2000 showed mean toxicities of 665–5300 MU/g (*n* = 10). These data prompted a toxicological surveillance of ribbon worms from other localities with different habitats in Japan, including Akkeshi Bay (Hokkaido) under stones on rocky intertidal beaches, as well as Otsuchi (Iwate) among calcareous tubes of serpulid polychaetes on rocky shores. Within twelve species of ribbon worms examined, only *C. simula* possessed extremely high toxicity. Therefore, *C. simula* appears to show generally high toxicity irrespective of their locality and habitat.

## 1. Introduction

Nemerteans are mostly marine, soft-bodied, vermiform invertebrates distributed worldwide, often inhabiting under rocks, among sessile organisms or within various sediments along coastal areas [1]. With approximately 1200 known species [2], they comprise the phylum Nemertea, which is classified into three subgroups, Palaeonemertea, Pilidiophora (=Heteronemertea + *Hubrechtella* and related forms) and Hoplonemertea [3,4,5]. They are generally carnivorous, with no morphological or behavioral means of protection against potential predators. Their food items so far studied include, among others, annelids, crustaceans and mollusks [6,7,8,9,10,11]. They rely on various toxic or noxious chemicals for their defense and predation [4,12]. The earliest report that nemerteans possessed toxins was by Bacq [13,14], who discovered two neurotoxins, each from (1) *Amphiporus lactifloreus* and *Drepanophorus crassus* and (2) *Lineus longissimus* and “*Lineus lacteus*” (=*Ramphogordius lacteus*). He suggested that these substances, which he called “amphiporine” and “némertine”, respectively, served in a defensive role, rather than being offensive venoms, such as those frequently associated with the prey captured by a characteristic eversible organ, the proboscis. This suggestion was supported by Kem [15,16,17] who reported that 70% of the total anabaseine present in the hoplonemertean, *Paranemertes peregrina*, was located in its integument. Species of *Amphiporus*, *Cerebratulus*, *Lineus* and *Tetrastemma* have also been found to contain other toxins, including neurotoxic polypeptides and pyridyl alkaloids [18,19]. Nemerteans are unique among metazoans in possessing an eversible proboscis, housed in a coelom-homologous [20,21], fluid-filled body cavity, the rhynchocoel. In marine, free-living nemerteans, the proboscis is used in prey capture. When within the range of a living prey, the proboscis is everted with explosive force and coils tightly around the prey’s body, which soon becomes inert or dead before eaten [22,23,24,25]. Therefore, it is likely that neurotoxins are generally involved in nemertean prey capture.

In 1998, during a toxicological surveillance of various marine fouling organisms in Hiroshima Bay, Japan, we found that palaeonemerteans in the genus *Cephalothrix* among sessile organisms on the shells of the cultured oysters, *Crassostrea gigas*, contained toxins with extremely strong paralytic activity [26]. The maximum toxicity (as tetrodotoxin, TTX) was 25,590 mouse units (MU) per gram for the whole body throughout the monitoring period [27,28]. This paralytic toxicity was identified to be caused by a high concentration of TTX, as described below.

Here, we focus on the toxicity of TTX-containing species of ribbon worms in Hiroshima Bay and review the current information, particularly compared with the toxicity of specimens from elsewhere.

## 2. Toxicological Surveillance of Ribbon Worms in Hiroshima Bay

**Figure 1:**
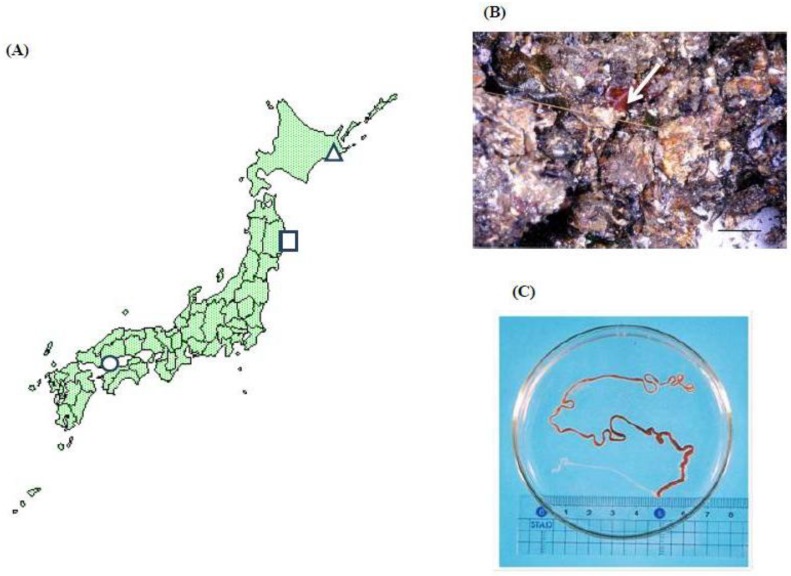
Sampling locations of ribbon worms in Japan. Hiroshima Bay (○), Otsuchi Bay (□) and Akkeshi Bay (Δ) are shown in the map (**A**); (**B**) Arrow indicates the ribbon worm *Cephalothrix simula* adherent to cultured oysters from Hiroshima Bay; scale bar: 10 cm. (**C**); *Cephalothrix simula* from Hiroshima Bay with the everted proboscis.

Figure 1 shows the map (A) showing collecting localities of ribbon worms (B, C) adhering to oysters cultured by hanging-culture method using floating rafts in Hiroshima Bay, Hiroshima Prefecture, which presents one of the major oyster culture areas in Japan. The paralytic toxicity of a species of ribbon worms (later identified as *Cephalothrix simula*), found on the surface of the shells of cultured oysters hanging onto floating culture raft in Hiroshima Bay, was examined between 1998 and 2005. Each time, 10 specimens were selected at random from the collected ribbon worms; each specimen was used for examination of toxicity. The toxicity score (MU/g) as TTX was expressed as the average of the 10 measurements.

**Figure 2:**
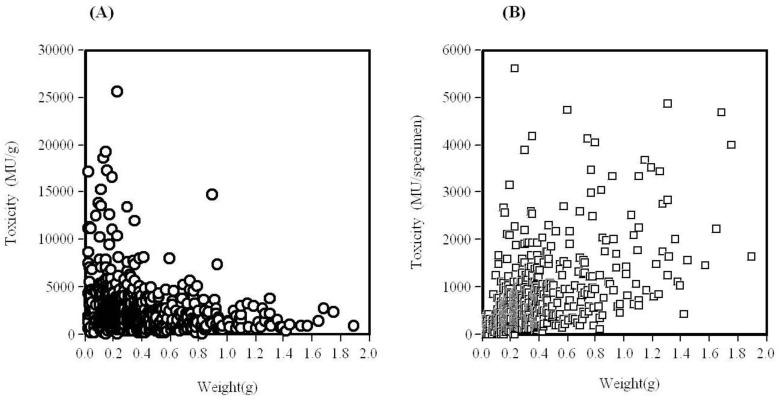
Toxicity of the ribbon worms *Cephalothrix simula* from Hiroshima Bay in 1998-2005. (**A**): relationship between toxicity (MU/g) and body weight, (**B**): relationship between toxicity (MU/specimen) and body weight.

A total of 764 specimens were collected and assayed for paralytic toxicity in mice throughout the season. Their weight of average is 0.36 ± 0.30 g per specimen. Their toxicity on average was 2584 ± 2560 MU/g. All the specimens assayed throughout the season were found to be toxic, and the toxicity scores ranged from 169 to 25,590 MU/g for the whole body (Figure 2). Specimens of *C*. *simula* were generally highly toxic, irrespective of the date of collection, though a wide individual variation of toxicity was evident (Figure 2). Approximately 80% of the individuals were ranked as “strongly toxic” (≥1000 MU/g). Forty-eight percent of the specimens possessed toxicity scores of more than 2000 MU/g. No clear relationship was observed between body weight and toxicity. The highest toxicity detected was 25,590 MU/g in a specimen collected on June 25, 1999. This equated to approximately 5631 MU. This value is approximately equivalent to half of the minimum lethal dose of TTX in human, which is reported to be 10,000 MU. Its toxicity exceeds the lethal dose for humans, equivalent to 2 mg of TTX. This lethal potency was approximately 51- or 47-times greater than the highest scores recorded for two species of ribbon worm, *Lineus fuscoviridis* and *Tubulanus punctatus*, inhabiting the surface of rocks or soft mud on the seashore in the Seto Inland Sea, Japan [29]. No paralytic toxicity was detected in the shucked meat of the oysters with shell fouled with the ribbon worms. From a food hygiene point of view, shucked oyster meat in itself was safe to eat. Fouling organisms on the shell are removed by washing in the drum in the factory of shucked meat of oysters. As the culturing of edible bivalves, such as oysters and scallops are flourishing industry in Japan, data on the distribution of toxic ribbon worms in other areas besides Hiroshima Bay is urgently needed. The same statements are true for the culturing of bivalves throughout the world. In this connection, it has been reported that TTX and related substances are present in the same species *Cephalothrix simula* (though erroneously identified and reported as *C. linearis*) in other part of the country, Shimoda [30]. 

**Figure 3:**
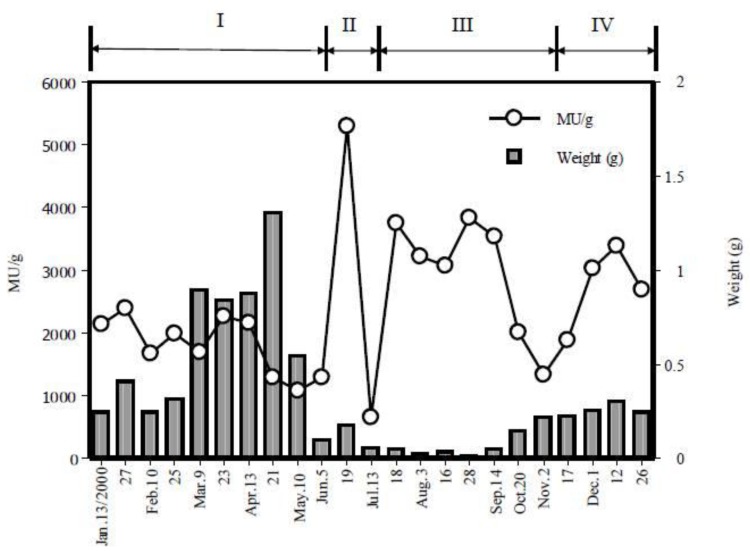
Seasonal variation of the average toxicity and body weight of the ribbon worm *Cephalothrix simula* from Hiroshima Bay in 2000. Seasonal variation of the toxicity was divided into four sections (I–IV).

Seasonal variations were observed in the lethal potency of *C. simula* in Hiroshima. Specimens collected on January 13, 2000 to December 26, 2000 showed mean toxicities of 665–5300 MU/g (*n* = 10). There were four periods of transition in toxicity of *C. simula* through a year as shown in Figure 3. Body weight increased from January to early summer (the latter half of April) and decreased thereafter up to September; then, it gradually increased.

## 3. Isolation of Main Toxic Component

### 3.1. Mouse Bioassay for Lethal Potency

Toxicity was examined by the standard bioassay method for TTX [28]. One mouse unit (MU) was defined as the amount of toxin, which killed a 18–20 g ddY strain male mouse in 30 min after intraperitoneal administration.

### 3.2. Column Chromatography

**Figure 4:**
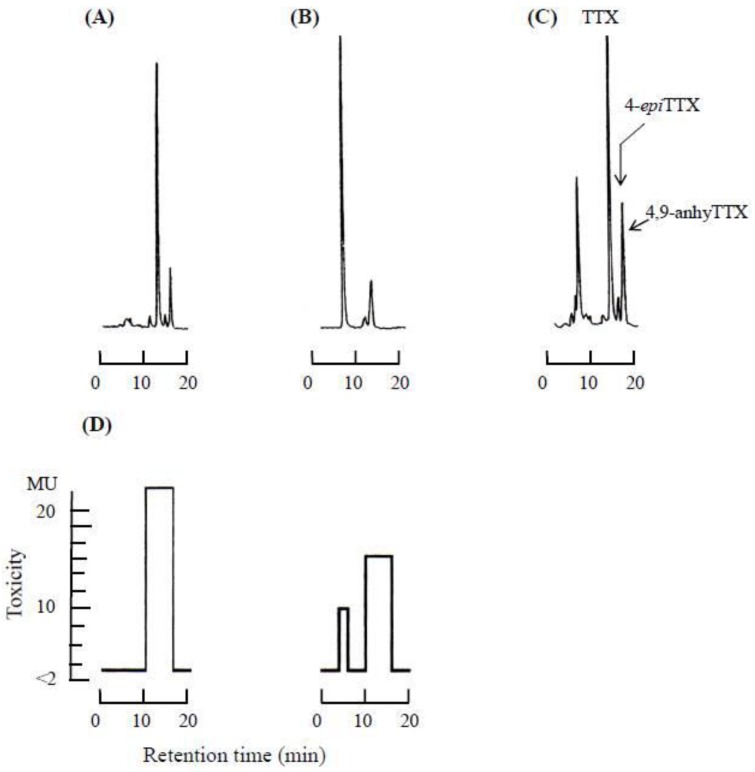
High performance liquid chromatography**-**fluorescence detection (HPLC-FLD) patterns (top) of fractions from the toxins contained in the ribbon worm, *Cephalothrix simula*, in an activated charcoal column chromatography. The bottom patterns represent the distribution of toxicity in HPLC chromatograms, as estimated by mouse bioassay. (**A**): fraction II bound on activated charcoal column; (**B**): fraction I unbound on activated charcoal column; (**C**): TTX standards; TDA (tetrodonic acid), TTX (tetrodotoxin), 4*epi*-TTX (4*epi*-tetrodotoxin) and 4,9-anhyTTX (4,9-anhydrotetrodotoxin); (**D**) Distribution of toxicity in the chromatograms of (**A**) and (**B**).

The specimens of ribbon worms (390 g) obtained during the survey were semi-defrosted and homogenized with three volumes of 1% AcOH in 80% MeOH for 3 min, then centrifuged. This operation was repeated two more times. The supernatants (total toxicity; 2,897,000 MU) were combined, concentrated under reduced pressure and defatted by shaking gently with approximately the same volume of chloroform several times. The aqueous layer (2,750,000 MU) was applied to anactivated charcoal column, and the adsorbed toxin was eluted with 1% AcOH in 20% EtOH after washing the column with distilled H_2_O. The water eluate (Fr.I; Fr = fraction I) and the eluate with 1% AcOH in 20% EtOH (Fr.II) was isolated. Each fraction was analyzed by high performance liquid chromatography (HPLC)-fluorescent detection (FLD) [31,32] (Figure 4). 

**Figure 5:**
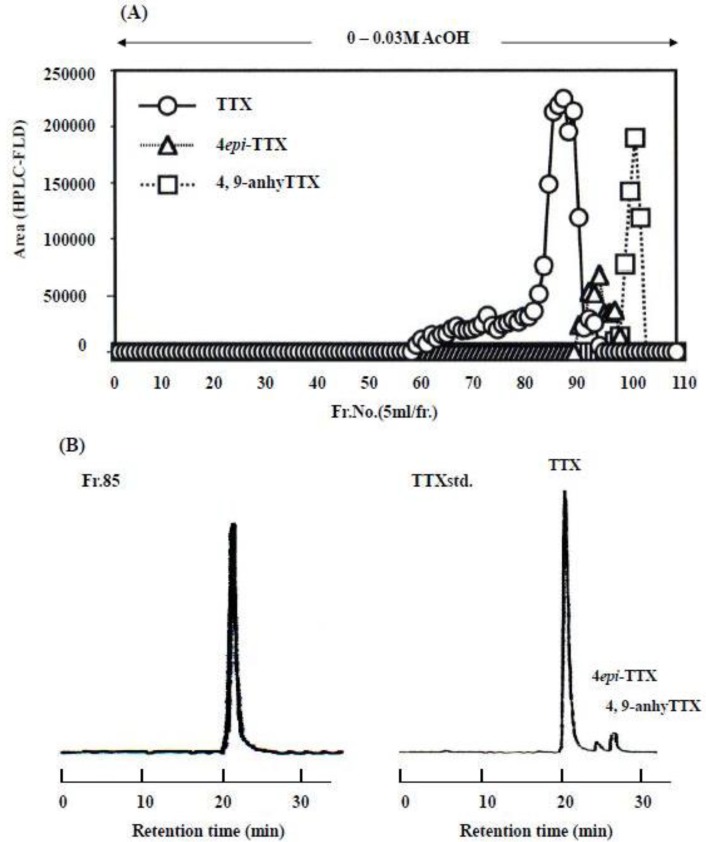
Elution profile of the ribbon worm *Cephalothrix simula* toxin from a Bio-Rex 70 column with linear gradient from 0 to 0.03 M acetic acid. (**A**): elution diagram; (**B**): toxin compositions contained in fraction Fr.85 by HPLC-FLD analysis; Fr.85 (left), Tetrodotoxin standards (right).

The main toxic fraction (Fr.II) was evaporated to dryness *in vacuo*. The resulting residue (total toxicity 2,433,000 MU; specific toxicity 99 MU/mg) was dissolved in a small amount of water, and the pH was adjusted to 5.5 with 1 N NaOH. This solution was applied to a Bio-Gel P-2 column (φ 3.5 × 100 cm). The column was washed with 3000 mL of water and then eluted with 2000 mL of 0.03 M AcOH. The toxicity was detected exclusively in the 0.03 M AcOH fraction. This fraction was concentrated to dryness under reduced pressure and the residue (3300 MU/mg) was dissolved in a small volume of water. The resulting solution was chromatographed on a Bio-Rex 70 (H^+^, φ 1.0 × 100 cm) column using a linear gradient of H_2_O and 0.03 M AcOH at the flow rate of 0.5 mL/min. The toxic fractions were monitored for TTX via the mouse bioassay and HPLC-FLD. The main toxic fractions (Fr.I; Fr.85–100) and minor fractions (Fr.II; Fr.50–84) were obtained and re-chromatographed in the same manner (Figure 5). 

The toxic fraction (Fr.I) thus obtained were freeze-dried and then dissolved in 0.5 mL of 1% AcOH. Approximately 2.0 mL of MeOH and 5.0 mL of diethyl ether were added to this solution, and the mixture was stored in the refrigerator overnight. During storage, stratified plate-like crystals appeared. The crystals were isolated by decantation and recrystallized, as that described above. Bio-Gel P-2 column chromatography was very effective, as the specific toxicity sharply increased from 99 to 3300 MU/mg. After recrystallization, the specific toxicity of this toxin increased to 3520 MU/mg. From the combined homogenates with the toxicity of roughly 7400 MU/g, approximately 25 mg of the stratified plate-like crystalline was obtained. Generally, the ribbon worm has a simple structure. Since pure crystals of TTX could be obtained from *C. simula* efficiently by the above-described series of chromatographies, *C. simula* is a promising source of TTX for use as a reagent in the fields of medicine and pharmacology. Previously, authentic specimens of TTX were typically prepared from pufferfish ovaries for use as reference standards, as reported in [33].

### 3.3. Instrumental Analysis

#### 3.3.1. Mass Spectrometry

A portion of the crystals was dissolved in a small amount of 1% AcOH and electrospray ionization-mass spectrometry (ESI-MS) was performed using a Hitachi M-1000. As the mobile phase, 50% methanol was used with a flow rate of 50 μL/min. The positive ion mass spectrum was measured.

#### 3.3.2. IR Spectrometry

A portion of the crystals was on a small KBr plate and the infrared (IR) spectrum was measured using a FT-IR spectrometer (Perkin Elmer, Spectrum 2000, Waltham, MA, USA) equipped with FT-IR microscope.

#### 3.3.3. Nuclear Magnetic Resonance Spectrometry

Five milligrams of the crystals was dissolved in 0.5 mL of 1% CD_3_COOD in D_2_O and placed in a test tube. ^1^H-NMR spectrum obtained with a 500 MHz JEOL JNM-500 spectrometer, using the methyl group proton of acetone as the internal standard.

#### 3.3.4. Gas Chromatography-Mass Spectrometry

The trimethylsilyl (TMS) derivative of 2-amino-6-hydroxymethyl-8-hydroxyquinazoline (C_9_ base), was derived from the crystals and authentic TTX by the procedure described previously [29,30,31,32,34]. Both TMS derivatives were submitted to a Hewlett Packard gas chromatograph (HP-5890-II) equipped with a mass spectrometer (AutoSpec, Micromass Inc., Manchester, UK). A column (φ 0.25 × 250 cm) of UB-5 (GL Sci., Tokyo, Japan) was used, and the temperature was raised from 180 to 250 °C at a rate of 5 °C/min. The ionization voltage was 70 eV and ion source temperature was kept at 200 °C. Scanning was carried out in the mass range of *m*/*z* 40–600 at 3 s intervals.

### 3.4. Results of the Instrumental Analysis

**Figure 6:**
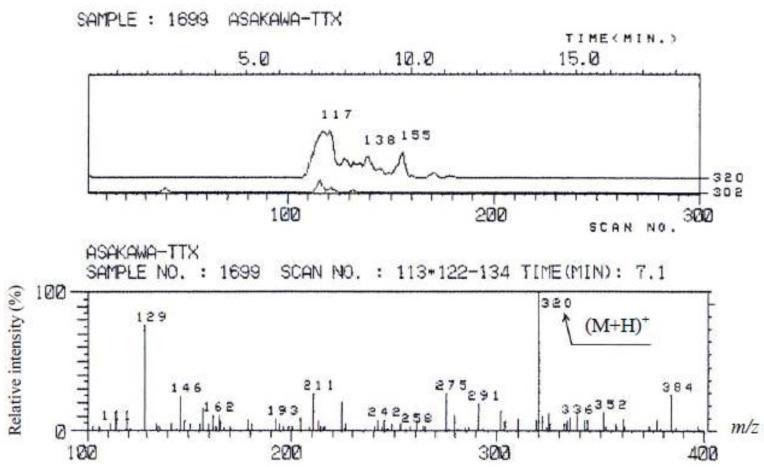
Liquid chromatography—mass spectrometry **(**LC-MS) of the toxin from the ribbon worm *Cephalothrix simula* from Hiroshima Bay. (**Upper**): mass chromatogram of the ribbon worm toxin (**Lower**): mass spectrum of the ribbon worm toxin.

The result of ESI-MS spectral analysis of toxins contained in *C. simula* is shown in Figure 6. The ESI mass spectrum showed an intense ion peak of (M + H)^+^ at *m*/*z* 320 and a weak ion peak of (M + H − H_2_O)^+^ at *m*/*z* 302. This mass spectrum agreed well with that of TTX [35,36]. The molecular weight thus determined was 319 for TTX, in accordance with its reported molecular weight. 

**Figure 7:**
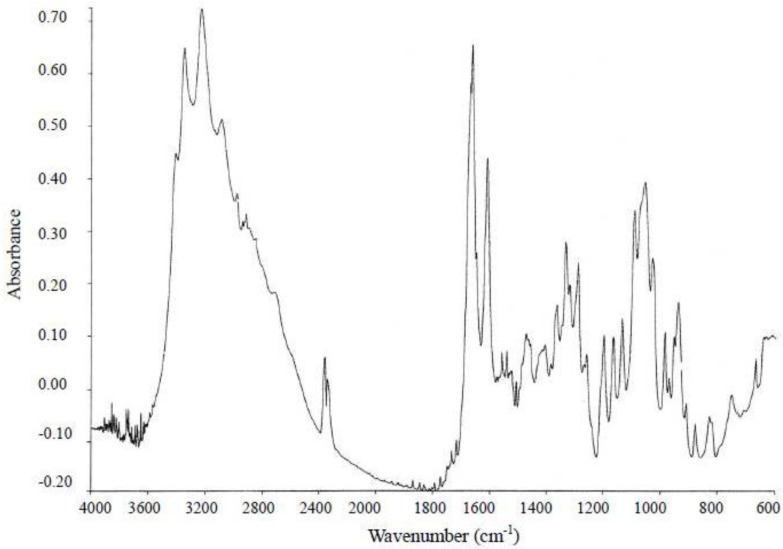
Infrared (IR) spectrum of the toxin isolated from the ribbon worm *Cephalothrix simula* from Hiroshima Bay. IR spectrum was taken on a FT-IR spectrometer (Perkin Elmer, Spectrum 2000,Waltham, MA, USA) equipped with FT-IR microscope.

As shown in Figure 7, the absorption band at 3353, 3235, 1666, 1612 and 1076 cm^−1^ were observed in infrared spectrum. This spectrum was indistinguishable from that of TTX reported [37]. The absorption around 2400 cm^−1^ derived from the existence of CO_2_ in the air. The absorption around 1800 cm^−1^ and 3600 to 4000 cm^−1^ is derived from the existence of H_2_O in the air. 

**Figure 8:**
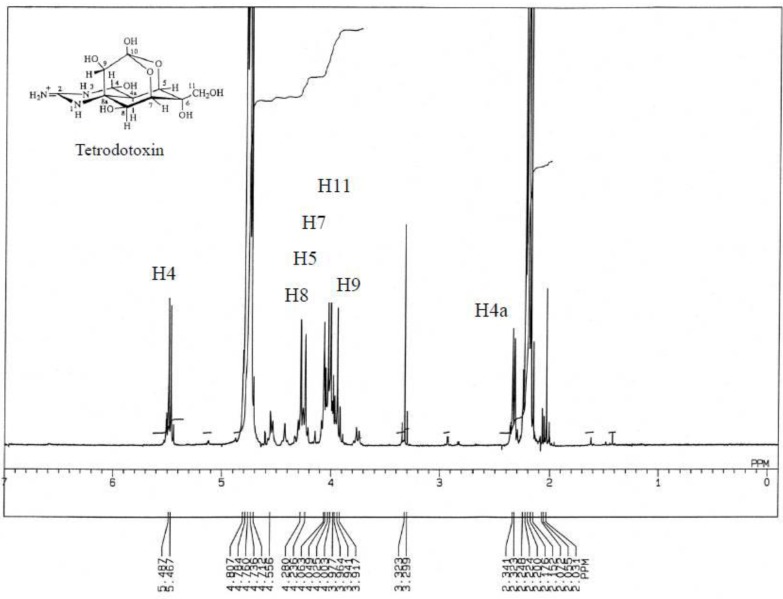
^1^H-NMR spectrum of the toxin isolated from the ribbon worm *Cephalothrix simula* from Hiroshima Bay. Five milligrams of HMT was dissolved in 0.5 mL of 1% CD_3_COOD in D_2_O and measured for ^1^H-NMR spectrum measured on a JEOL JNM-500 NMR spectrometer, using acetone as the internal standard.

**Figure 9:**
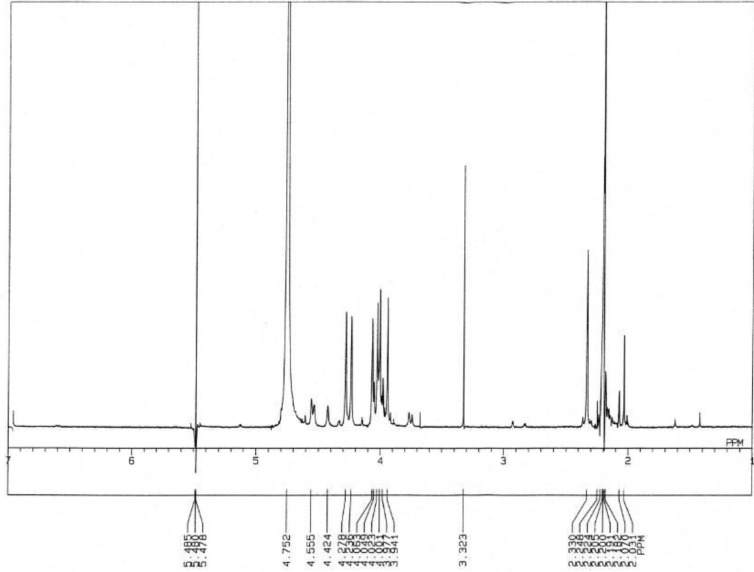
^1^H-NMR spectrum of the toxin isolated from the ribbon worm *Cephalothrix simula* from Hiroshima Bay by means of irradiation at C_4_-H. Five milligrams of HMT was dissolved in 0.5 mL of 1% CD_3_COOD in D_2_O and measured for ^1^H-NMR spectrum measured on a JEOL JNM-500 NMR spectrometer, using acetone as the internal standard.

**Figure 10:**
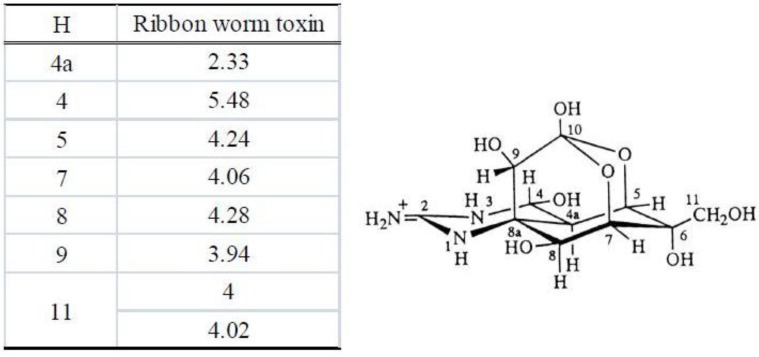
Comparison of 1H-NMR spectral data of the toxin isolated from the ribbon worm *Cephalothrix simula* from Hiroshima Bay, along with structure of TTX.

As shown in Figure 8, ^1^H-NMR spectrum of toxins contained in *C. simula* exhibited a singlet at 2.20 ppm (CH_3_COCH_3_), a doublet centered at 2.33 ppm (*J* = 10.0 Hz), a large proton peak at 4.76 ppm (HDO) and a doublet centered at 5.48 ppm (*J* = 10.0Hz). The pair of doublets around 2.33 and 5.48 ppm, which are the hallmarks of TTX and are assigned to H-4a and H-4, respectively, were confirmed to be coupled with each other by double irradiation (Figure 9) [38,39]. These results agree well with the corresponding data of TTX. The signals at 4.24, 4.06, 4.28, 3.94, 4.00 and 4.02 ppm are assigned to H-5, H-7, H-8, H-9 and H-11 respectively (Figure 10). 

**Figure 11:**
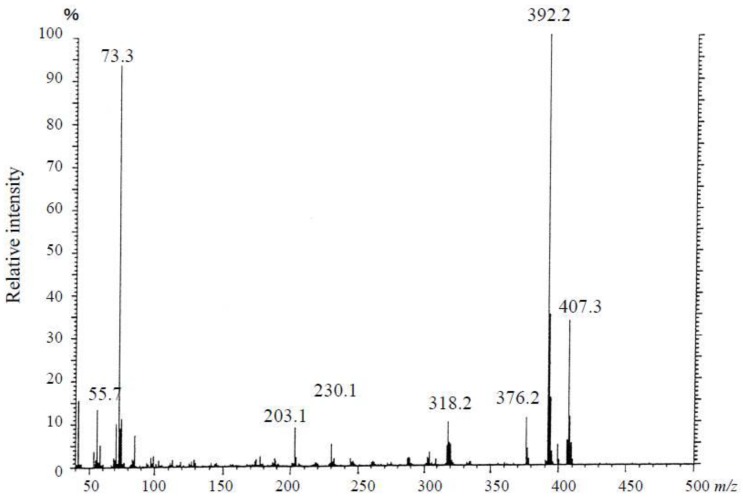
Mass spectrum of the trimethylsilyl (TMS) derivative of alkali-hydrolyzed toxin from the ribbon worm *Cephalothrix simula* from Hiroshima Bay.

**Figure 12:**
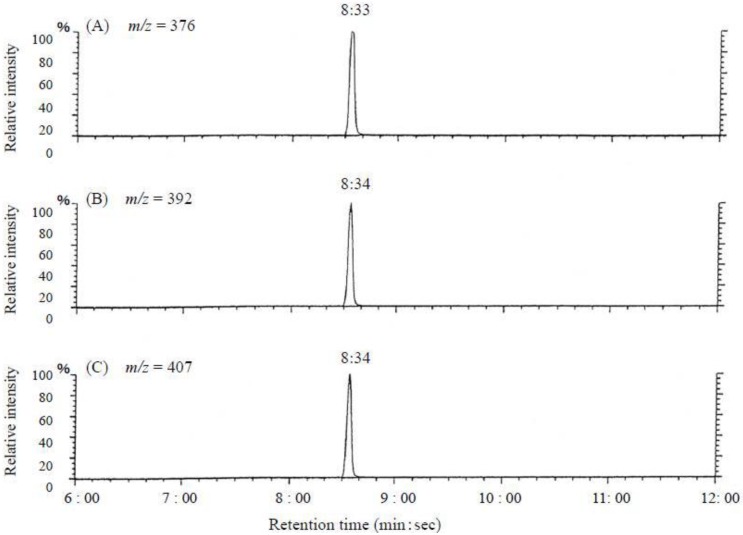
Selected ion-monitored mass chromatograms of the trimethylsilyl (TMS) derivative from alkali-hydrolyzed toxin from the ribbon worm *Cephalothrix simula* from Hiroshima Bay. (**A**) *m*/*z* = 392 (**B**) *m*/*z* = 407 (**C**) *m*/*z* = 376.

In the selected ion-monitored mass chromatogram of the trimethylsilyl (TMS) derivatives of alkali-hydrolyzed toxins prepared from *C*. *simula*, mass fragment ion peaks at *m/z* 376, 392 and 407, which are characteristic of the quinazoline skeleton (C_9_ base), appeared at almost the same retention times (8:33 and 8:34; minute:second, respectively), as from the TMS-C_9_ base derived from authentic TTX (Figure 11, Figure 12). These facts indicate that the present toxin also contained the quinazoline skeleton specific to TTX [29,30,31,32,34]. These results allowed us to conclude that this toxin, a major component of the paralytic toxins contained in this species of the genus of *C. simula*, was nothing else than TTX.

## 4. Distribution of TTX in *C. simula* from Hiroshima Bay

The nemertines are a phylum of carnivores capable of capturing and ingesting prey several times their own size. The tissue and species distribution of TTX should be investigated in order to understand better the function of this toxin in the nemertine phylum. Nemerteans are unique because they possess an eversible proboscis, housed in a coelom-homologous, fluid-filled body cavity, called the rhynchocoel. They possess a characteristic eversible organ, the proboscis, from which venom released is in prey capture. The nemertean proboscis is a digestive-tract-independent, essentially musculo-glandular tubular structure and lies, when retracted, in the fluid-filled proboscis chamber, the rhynchocoel, situated dorsal to the alimentary canal. The proboscis is everted through a pore at the tip of the head, the rhynchostome. The most common food sources for adult nemerteans are polychaetes, as well as small crustaceans and mollusks and, occasionally, fish [40]. The proboscis, when used in prey capture, is everted with explosive force and coils tightly around the prey’s body. It was observed that the living prey caught by either *Cephalothrix linearis* or *Cephalothrix bioculata* became inert and apparently lifeless in 30 s before the nemertean swallowed down the prey [23]. The proboscis epithelium of nemerteans in the genus *Cephalothrix* contains tiny rod-like structures, or pseudocnides, which are thought to act as either weapons to cause wounds in the body of a potential prey, through which toxic substances could be poured or grips that may assist the proboscis in its mechanical hold on the prey. The histological distribution of TTX in *C. simula* from Hiroshima Bay was examined by immunostaining research using anti-TTX antibody [41,42]. The results showed that in this species TTX was located in the following: (1) vesicles apically arranged in the epidermal cells; (2) vesicles in the basal position of intestinal epithelial cells abutting lateral blood vessels and (3) glandular cells in the proboscis epithelium. This arrangement indicates that TTX in the prey organism is taken in through intestinal cells, transferred to the blood vessels and spreads throughout the body, before being actively transported to both the epidermal cells and the proboscis glandular cells that are used in defense and offense mechanisms, respectively. Ribbon worms have a long thread-like proboscis, which can extend explosively on stimulation, as also observed by the present authors. The worms actively seek out and capture their prey using their long proboscis, which acts as an exploratory organ, as well as a device for snaring the prey. Therefore, ribbon worms may utilize their proboscis as a defensive and/or offensive weapon. 

## 5. Toxicological Surveillance of the Nemertean Species from Other Locations

A literature-based taxonomic catalogue of the nemertean species (phylum Nemertea) reported from Japanese waters is provided, listing 19 families, 45 genera and 120 species, as valid [5]. Here, attempts were made to survey toxicity of ribbon worms from other locations. From two stations of Otsuchi Bay, Iwate Prefecture, and Akkeshi Bay, Hokkaido, in addition to Hiroshima Bay, ribbon worms were collected to examine their toxicity (Figure 1). 

In Akkeshi Bay, ten species of ribbon worms were collected in July, 2000, 2008 and 2009 (Table 1). High toxicity was detected only in *Cephalothrix simula*, and TTX was clearly detected by HPLC-FID and GC-MS analysis. In spite of nontoxic specimens, in HPLC-FLD and GC-MS analysis, TTX and its derivatives were detected.

**Table 1:** Toxicity of ribbon worms collected from Akkeshi Bay, Hokkaido.

Ribbon worms	Date of	No. of	Weight (g)	Toxicity (MU/g )	HPLC-FLD			GC-MS
	collection	specimens	(Mean ± S.D.)	(Mean ± S.D.)	TTX	4epi-TTX	4,9-anhyTTX	
*Nemertellina yamaokai*	30 July 2000	16	0.04 *^1^	ND	−	−	−	−
*Micrura akkeshiensis*	30 July 2000	3	0.02 *^1^	ND	*^2^	*^2^	*^2^	−
*Malacobdella japonica*	30 July 2000	3	0.03 ± 0.01	ND	−	−	±	−
*Tetrastemma nigrifrons*	30 July 2000	3	0.03 ± 0.02	ND	−	−	−	−
*Tetrastemma stimpsoni*	30 July 2000	5	0.02 *^1^	ND	*^2^	*^2^	*^2^	*^2^
*Amphiporus* sp.	30 July 2000	2	0.03 ± 0.01	ND	+	−	−	−
*Lineus bilineatus*	30 July 2000	12	0.03 *^1^	ND	−	−	−	−
*Cephalothrix simula*	30 July 2000	3	0.12 ± 0.06	1223 ± 97	+	±	+	+
*Lineus torquatus*	30 July 2000	1	4.23	ND	+	±	+	+
*Lineus alborostratus*	30 July 2000	1	1.81	ND	+	+	+	+
*Cephalothrix simula*	9 April 2008	6	0.17 ± 0.11	2958 ± 2483	+	+	+	+
	1 July 2008	11	0.42 ± 0.22	1226 ± 614	+	+	+	+
	18 October 2008	8	0.29 ± 0.21	1683 ± 2220	+	+	+	+
	11 January 2009	11	0.21 ± 0.11	579 ± 343	+	+	+	+

*^1^: combined specimen; *^2^: not tested; ND: not detected; +: Clearly detected; ±: Difficult to detect; −: Not detected.

On the other hand, in Otsuchi Bay, Iwate Prefecture, three species of ribbon worm were collected in September, 2000 (Table 2). TTX was detected in *C*. *simula*. However, though toxicity was not detected in *Nipponnemertes punctatula*, TTX and its derivatives was detected clearly by HPLC-FID and GC-MS analysis. 

**Table 2:** Toxicity of ribbon worms collected from Otsuchi Bay, Iwate Prefecture.

Ribbon worms	Date of collection	No. of specimens	Weight (g)	Toxicity (MU/g )	HPLC-FLD			GC-MS
			(Mean ± S.D.)	(Mean ± S.D.)	TTX	4epi-TTX	4,9-anhyTTX	
*Cephalothrix simula*	26 September 2000	2	0.25 *	2021 ± 247	+	±	+	+
*Nipponnemertes punctatula*	28 September 2000	2	0.03 ± 0.01	ND	+	±	±	+
*Cephalothrix simula*	28 September 2000	2	0.07 *	1708 ± 279	+	±	±	+
unknown specimen	28 September 2000	12	0.22 *	ND	−	−	+	+

*: combined specimen; ND: not detected; +: Clearly detected; ±: Difficult to detect; −: Not detected.

To compare the toxicity of *C. simula* from Japan, the toxicity of *Cephalothrix* from Qingdao, China, was examined in March, 2009. In the sixteen specimens of *Cephalothrix*, high toxicity was detected in the same manner of Japanese specimens [43].

## 6. Origin of TTX Compounds in the Ribbon Worm

It is known that some intestinal bacteria of TTX-bearing animals, as well as some marine bacteria, are endowed with a TTX-producing ability [44,45]. This, along with various findings so far obtained, seem to indicate the involvement of the following mechanism in the toxification of TTX-bearing animals. First, some TTX-producing marine bacteria enter and inhabit the intestines of invertebrates of lower-strata in the food chain. These bacteria produce TTX and/or related substances, which are accumulated in the hosts and then transferred to organisms of middle strata through predation. The physiological relationship between those bacteria and lower-strata invertebrates is still unclear. Finally, carnivorous animals, whether invertebrates or vertebrates as represented by pufferfish, feed on these toxic lower strata invertebrates, accumulating TTX and/or its related substances efficiently. A portion of the toxin may come directly from TTX-producing bacteria inhabiting the intestines or even from the sediment.

So the TTX infestation mechanism in *C. simula* may involve symbiotic and parasitic microorganisms, which produce TTX, though the possibility that TTX is accumulated in them through food chain cannot be fully excluded. Intestinal contents of *C. simula* collected in Hiroshima Bay, Hiroshima Prefecture, Japan, in May, 2003, were examined for bacterial flora. From the extract of one strain with 0.1% AcOH, a little amount of TTX was detected by the results of LC-TOFMS and GC-MS, along with mouse bioassay [46]. In this connection, a marine bacterium, provisionally identified as *Vibrio alginolyticus*, which is associated with TTX production, was isolated from several species of marine nemerteans [47]. Carrol *et al.* showed that a relationship of *Vibrio* bacteria, probably *Vibrio alginolyticus*, and the synthesis of TTX-like substances in seven species of British nemerteans [48].

## 7. General Discussion

Tetrodotoxin (TTX), a low molecular weight neurotoxin, which was believed to occur exclusively in the ovaries and livers of pufferfish (Tetraodontidae) [33,37], has been found in representatives of several different marine phyla. In 1964, Mosher *et al.* detected TTX in the California newt, which was the first organism to be identified as containing TTX other than pufferfish [49]. Since then, the toxin has been detected in a tropical goby [50], Costa Rican frogs [38] and frogs in the Brazilian Atlantic rain forest [51], the blue-ringed octopus [39,52] and several species of carnivorous gastropods, such as trumpet shell [34], ivory shell [53], frog shell [54] and the grey side-gilled sea slug [55]. In addition, some species of starfish on which these gastropods feed also contained TTX [56]. Toxic crabs [31,57,58], flatworms [59,60,61], a horseshoe crab [62], ribbon worms and arrow worms [63] have also been identified as TTX-containing animals. The two species of Japanese nemerteans, the palaeonemertean *Tubulanus punctatus* and the heteronemertean *Lineus fuscoviridis*, also contained TTX [29]. A third species, the palaeconemertean *Cephalothrix linearis*, was subsequently found to contain TTX and related substances [30]. 

Nemertines are a phylum of carnivorous marine worms that possess a variety of alkaloidal, peptidic or proteinaceous toxins that serve as chemical defenses against potential predators [8]. In this connection, structure and membrane actions of a marine worm protein cytolysin, called “Cerebratulus toxin A-III”, was reported [64].

This time, extremely high levels of these toxins have also been recorded from a *Cephalothrix* *simula*, which was found among cultured oysters in Hiroshima Bay. Of the TTX-bearing animals, our specimens of ribbon worms (“akahana-himomushi” in Japanese) adherent to the cultured oyster *Crassostrea gigas* hanging onto floating culture rafts, were found to be extremely toxic and to contain TTX, during surveillance of the toxicity of various marine fouling organisms in Hiroshima Bay, Hiroshima Prefecture, which is one of the largest oyster culture areas in Japan. In these analyses, the toxicity was examined on each ribbon worm specimen by the standard bioassay method for TTX. Ribbon worm specimens were collected in Hiroshima Bay between November and May from 1998 to 2005, approximately every two weeks during the harvest time for cultured oysters. A total of 764 specimens were collected and assayed for toxicity. All specimens that were assayed throughout the season covered found to be toxic and the toxicity scores ranged from 169 to 25,590 MU/g (Figure 4, Figure 5). The ratio of strongly toxic (more than 1000 MU/g) specimens to the total number of specimens was 80%. Furthermore, the percentage of specimens possessing toxicity scores higher than 2000 MU/g to the total was high (48%). The highest toxicity detected was 25,590 MU/g from a specimen collected on June 25 (1999). The total toxicity for this sample was approximately calculated to be 5631 MU, which is approximately equivalent to half of the minimum lethal dose of TTX in humans, which is reported to be 10,000 MU. 

The occurrence of TTX in the ribbon worm is of great interest because of its high abundance. TTX has been shown to be present not only in the puffer fish, but also in a variety of vertebrates and invertebrates. The mechanism of the induction of toxicity in TTX-containing animals and the dynamic state of TTX and its related compounds has not been completely described. There is no phylogenetic relationship among these TTX-containing animals. The origin of TTX compounds in the ribbon worm also remains to be elucidated. The wide individual variations in toxicity of the ribbon worms, which are carnivorous, suggested that TTX is exogenous. Though the life history of nemertean worms has not been clarified yet, these experiments may prompt investigation of TTX-toxification mechanism of the ribbon worm *C.* *simula* from Hiroshima Bay.
